# Seroprevalence of Fascioliasis in Patients Referred to the Reference Laboratory in Urmia, Northwest Iran

**DOI:** 10.1155/japr/2019223

**Published:** 2026-07-06

**Authors:** Fatemeh Ramzi, Zahra Ghahvechi Amiri, Diana Oladzad Abbas Abadi, Kambiz Diba, Yahya Azadeh, Mahya Zolfaghar Ghoshooni, Shiva Zeinali, Rasool Jafari

**Affiliations:** ^1^ Student Research Committee, Urmia University of Medical Sciences, Urmia, Iran, umsu.ac.ir; ^2^ Department of Medical Parasitology and Mycology, School of Medicine, Urmia University of Medical Sciences, Urmia, Iran, umsu.ac.ir; ^3^ Department of Medical Laboratory Sciences, School of Allied Medical Sciences, Urmia University of Medical Sciences, Urmia, Iran, umsu.ac.ir

**Keywords:** *Fasciola*, fascioliasis, Iran, prevalence, Urmia

## Abstract

**Background:**

Iran is one of the most important foci of human fascioliasis. West Azerbaijan Province is known for its traditional rural livestock husbandry, supported by many rivers. This environment could facilitate the transmission of fascioliasis between animals and humans. Thus, the present study investigated the seroprevalence of fascioliasis in patients referred to the Reference Laboratory in Urmia, Northwest Iran.

**Materials and Methods:**

In this cross‐sectional study, 919 sera were collected from patients referred to the Reference Laboratory of Urmia University of Medical Sciences from January to June 2023. The samples were examined by Enzyme‐Linked Immunosorbent Assay(ELISA) to detect IgG antibodies against *Fasciola hepatica*. Demographic information and symptoms of the participants were collected through a questionnaire.

**Results:**

Of 919 sera tested, 532 (57.9%) and 387 (42.1%) were women and men, respectively. Among 919 subjects, three (0.326%) were seropositive for fascioliasis. Of the three positive cases, two were women aged 31 and 79 years, and one was a 60‐year‐old man; all were rural residents. All three positive cases had livestock around them in the village. Because of the small number of positive cases, further statistical analysis was not performed.

**Conclusion:**

The present findings suggest the presence of human fascioliasis in the Urmia District, West Azerbaijan Province, Northwest Iran, especially in rural areas, with a low prevalence. Therefore, the health care systems require educating the residents of the villages about fascioliasis, the route of its transmission, and strategies required for prevention.

## 1. Introduction

Fascioliasis is a zoonotic infectious parasitic disease affecting both animals and humans, caused by two species of liver flukes, *Fasciola hepatica* and *Fasciola gigantica*, which impose a burden on public health*. F. gigantica* is more prevalent in Asia and Africa, whereas infections with *F. hepatica* have been reported on almost all continents; therefore, it has a wide geographical distribution. Fascioliasis can impact meat and milk production, leading to liver damage and reduced livestock fertility, thereby causing significant harm to the livestock industry [1].

*F. hepatica* has a wide geographical distribution and has been reported from many regions all around the world [2]. In addition, 12,500 deaths from fascioliasis were recorded around Lake Titicaca, in the Andes Mountains on the border of Bolivia and Peru, as one of the highest prevalences of human infections in the world [3].

In Asia, human fascioliasis is primarily found in Iran in the Middle East, and to a lesser extent in Vietnam, a Southeast Asian country. Studies conducted between 1997 and 2000 have identified more than 500 human cases of fascioliasis in Vietnam [2]. A recent serological survey estimated a remarkably high prevalence of *Fasciola* infection in Vietnam at 11.1% [4]. The World Health Organization (WHO) has recently included Iran among six countries known for serious problems with fascioliasis [5]. In South Asia, several cases of fascioliasis have been identified in India and Afghanistan [2]. Punjab Province of Pakistan has lately been known as an endemic region of human fascioliasis, and in other regions of this country, infection in children has been reported [6, 7]. Another important focus of human fascioliasis infection was reported in China in 2013 [2].

Most cases of human fascioliasis and outbreaks in Iran are found in the northern parts of the country, particularly Gilan Province and along the Caspian Sea. The largest outbreak occurred in 1989 and involved approximately 10,000 cases from Bandar Anzali and 2465 from the shores of the Caspian Sea. The second outbreak happened about 10 years later and resulted in 5000 infections in the same region [5]. Today, the disease is reported across most geographical regions of the country, particularly in the provinces of Gilan, Mazandaran, Kermanshah, and Isfahan [8].

Animal husbandry and agriculture are common and important occupations in the villages of West Azerbaijan Province. In Iran, rural men and women take their livestock for grazing, far away from their place of residence, to green lands with springs and rivers. If the grazing livestock are infected by *Fasciola* spp. and there is a suitable intermediate snail host, they can contaminate the surrounding water and vegetables [9, 10].

Previous studies have shown that fascioliasis, particularly *F. hepatica* infection, is prevalent among livestock in West Azerbaijan Province [11, 12]. However, no comprehensive study has evaluated human fascioliasis in this region. Therefore, there is limited information and a knowledge gap regarding the seroprevalence of the infection and its high‐risk areas in West Azerbaijan Province. Accordingly, the present study is aimed at investigating the seroprevalence of human fascioliasis among patients referred to the Reference Laboratory in Urmia.

## 2. Methods

### 2.1. Geographical Location

West Azerbaijan Province is situated in the northwest region of Iran, positioned between latitudes 35° 71 ^′^ 58 ^″^ N to 39° 46 ^′^ N, and longitudes 44° 3 ^′^ E to 47° 23 ^′^ E. It shares its borders with four countries: Armenia, the Nakhchivan Autonomous Republic of Azerbaijan, Iraq, and Türkiye [13]. This region is known for its diverse landscape, featuring forest steppes and a variety of climates, such as the Mediterranean hot summer climate, coastal Mediterranean climate, and cold winter climate [14]. Geographically, it includes mountainous areas along the borders of Iraq and Turkey, plains adjacent to the Aras River and other waterways, and the shores of Lake Urmia [13]. The province covers an area of about 4705/316 km^2^ (including Lake Urmia), and as per the 2016 census, the province has a population of 3,265,219 [15].

### 2.2. Study Population and Sampling

A total of 919 sera were collected from patients referred to the Reference Laboratory in Urmia (affiliated with Urmia University of Medical Sciences), West Azerbaijan Province, northwestern Iran, from January to June 2023. The sampling method was consecutive convenience sampling, and the humans studied were referred to the reference laboratory for other reasons, rather than fascioliasis, such as laboratory tests or for stool examination to get a food handler′s license.

### 2.3. Anti‐*Fasciola* IgG ELISA

The collected sera were tested for the presence of anti‐*Fasciola hepatica* IgG using a *Fasciola* ELISA kit according to the manufacturer′s manual (Pishtaz Teb, Iran). This qualitative kit uses *F. hepatica* antigen‐coated wells to detect anti‐*Fasciola* IgG. The test results are based on a cutoff, providing only positive or negative results; there is no measurement of IgG concentration or borderline results. The results were interpreted as follows: First, the OD of blank wells was subtracted from all ODs; then the cutoffs for each testing run were calculated separately as the OD of the negative control plus 0.25, with values above considered positive and those below negative. According to the kit′s manual, it has a sensitivity of 92%, specificity of 93%, accuracy of 93%, and a correlation of 97% for human fascioliasis, with cross‐reaction only occurring with schistosomiasis, which was eliminated from Iran years ago [16].

### 2.4. Demographics and Risk Factors

For each participant, a questionnaire was completed, gathering demographic information, including place of residence, consumption of aquatic wild vegetables, washing methods for vegetables, drinking water sources, proximity to animal husbandry, and symptoms through interviews.

### 2.5. GIS Map

The GIS map was built by ArcGIS Version 10.1 software to illustrate the exact location of positive cases.

### 2.6. Ethical Considerations

This study was approved by the Ethics Committee of Urmia University of Medical Sciences (Research Code: 3038; Ethical Code: IR.UMSU.REC.1401.156). All participants were informed about the study, and a signed consent was obtained from each one.

## 3. Results

In this study, 919 sera were tested, all of which were referred from the Urmia District. The mean age of the participants was 41.98 ± 17.94 years (ranging from 1 to 92). By gender, the participants included 532 (57.9%) women and 387 (42.1%) men, with mean ages of 41.88 ± 17.14 and 42.12 ± 19 years, respectively. No serum sample showed an OD near the cutoff, and among the 919 cases, three (0.326%) were positive: two women aged 31 and 79, and one man aged 60. The mean age of positive cases was 57.33 ± 1334 (Std). With only three cases being positive, statistical analysis 1 has low statistical power and cannot be judged. Table 1 shows cross tabulation of all collected demographic variables and risk factors.

**Table 1:** Cross tabulation of seropositivity against *Fasciola* among gathered demographic information and risk factors in studied humans in the Urmia District.

		Anti‐*Fasciola* IgG		Total	OR	95 CI	*p*
		Positive	Negative				
**Sex**	Female	2 (0.4)	530 (99.6)	532	1.457	0.132–16.121	0.617
	Male	1 (0.3)	386 (99.7)	387	1	—	
**Residential**	Urban	0 (0)	277 (100)	277	—	—	0.558
	Rural	3 (0.5)	639 (99.5)	642	—	—	
**Livestock husbandry**	Yes	1 (0.4)	250 (99.6)	251	1.332	0.12–14.754	0.616
	No	2 (0.3)	666 (99.7)	668	1	—	
**Vegetable**	Bought	2 (0.5)	374 (99.5)	376	2.898	0.262–32.08	0.571
	Self‐cultivated	1 (0.2)	542 (99.8)	543	1	—	
**Education**	University	0 (0)	82 (100)	82	—	—	0.645
	Guidance school	0 (0)	181 (100)	181	—	—	
	High school	1 (0.8)	132 (99.2)	133	—	—	
	Elementary school	0 (0)	232 (100)	232	—	—	
	Illiterate	2 (0.7)	289 (99.3)	291	—	—	
**Water source**	Tap water	3 (0.4)	825 (99.6)	828	—	—	1
	Spring water	0 (0)	39 (100)	39	—	—	
	Well water	0 (0)	52 (100)	52	—	—	
**Vegetable wash**	Salt‐water	3 (1.2)	247 (98.8)	250	—	—	0.98
	Detergent	0 (0)	103 (100)	103	—	—	
	Vinegar‐water	0 (0)	89 (100)	89	—	—	
	Water	0	477	477	—	—	
**Symptoms**	URQ pain	0 (0)	24 (100)	24	—	—	0.034
	Chest pain	1 (10)	9 (90)	10	—	—	
	No sign	2 (0.2)	883 (99.8)	885	—	—	
**Total**		3 (0.3)	916 (99.7)	919	—	—	

**Figure 1:**
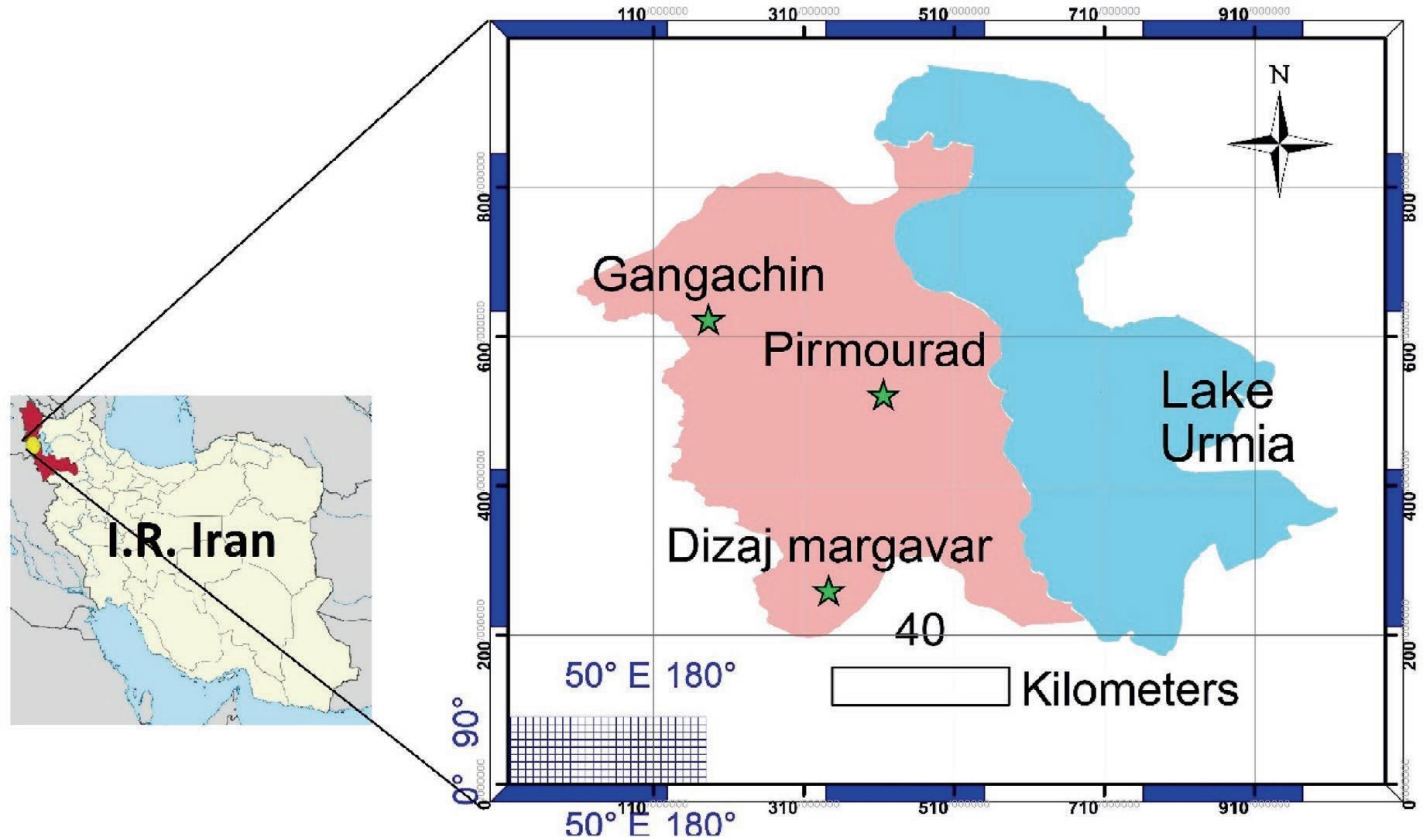
GIS map of location of the three seropositive cases of fascioliasis in the Urmia district, West Azerbaijan Province, Northwest Iran.

All three positive cases originated from rural areas where they had contact with livestock (their own or other livestock in the village) (Table 1). The locations of the three positive patients are shown in Figure 1.

## 4. Discussion

Herein, anti‐*Fasciola* IgG was positive in 0.326% out of a considerable population (919 humans), and fascioliasis‐related clinical symptoms were not observed in seropositive ones. A major limitation of the study was the inability to follow‐up patients for confirmatory tests beyond serology. Therefore, the ELISA results should be interpreted cautiously, as seropositivity may reflect either current or previous exposure to *Fasciola* spp.

In the present study, all three positive patients for fascioliasis consumed raw vegetables. However, in Iran, consuming different raw vegetables as salad is popular, and most people eat green salad. This observation aligns with studies conducted in Meshkin‐Shahr and Yasuj, showing no significant difference in vegetable consumption [17, 18]. In this regard, many recent investigations have shown that consuming fresh and local aquatic vegetables is a significant factor in infections with the parasite [9]. Ashrafi et al. [19] reported that Delar (green salt in Persian) and processed olives with vegetables (Zeytoon‐Parvardeh) are two important sources of human fascioliasis in northern Iran. The consumption of aromatic plants such as *Eryngium* spp. and *Mentha* spp., whether eaten fresh or prepared in various popular pastes or appetizers, has been identified as the most probable source of human infections in the endemic areas of Gilan Province [20, 21].

In South Asia, several cases of fascioliasis infection have been identified in India, Afghanistan [2], Pakistan [6, 7], Vietnam [2], and China [2]. In Iran, the Caspian sea basin is a well‐known region for endemic fascioliasis; however, it is also endemic in other regions of the country, granted in low prevalence such as the present study. The low prevalence of human fascioliasis is not special to Urmia, but in almost all general population‐based studies, the reporte prevalence is low [9, 17, 21–24]. The low prevalence reported in the present study may be reflected by several environmental and parasite‐related factors, such as a suitable intermediate snail environment for the intermediate host, and the parasite′s life cycle and transmission routes to humans [2], as well as the demographic characteristics of the tested population as in the present study. Fascioliasis transmission to humans occurs almost by eating raw, aquatic, self‐grown edible vegetables and unsafe water [2, 19]. This may not occur routinely for every human because not all aquatic vegetables are edible and not all humans eat them; on the other hand, most areas in Iran, even villages, have safe water these days.

Considering affected age, in the present study, the mean age of seropositive humans was 57 years ranging from 31 to 79. Contrary to our findings, in Bandar Anzali, North of Iran, most positive cases of human fascioliasis have been reported from people under 35 years of age [25]. Likewise, the prevalence of the disease in the age group under 20 years in the north of the country was significantly higher, as compared with other age groups [21]. In Meshkin‐Shahr in Northwest Iran, the highest rate of fascioliasis infection was related to the age group of 40–49 years [17]. In Lorestan province, the seroprevalence of human fascioliasis was estimated at 1.3% in people over 60 years of age [22]. In a report from Gorgan province, all seropositive cases were over 60 years of age [8]. Differences in the age distribution reported among studies may reflect regional variations in exposure and food habits; however, the small number of seropositive cases in the present study limits interpretation.

The present study provides serological evidence suggesting exposure to *Fasciola* spp. in Urmia District, West Azerbaijan Province, Northwest Iran. The prevalence is low, similar to other parts of Iran, and all seropositive cases were from rural areas. To our knowledge, this is the first report of human fascioliasis in West Azerbaijan Province.

## 5. Conclusion

The findings indicate that human exposure to *Fasciola* spp. occurs in this region, particularly in rural areas of West Azerbaijan Province, Northwest Iran, though at a considerably low prevalence, close or slightly lower than most reports from various areas in the country. The disease remains a health concern, particularly in rural communities. Public health initiatives should educate residents about transmission routes and preventive strategies. Further studies are needed to identify other potential endemic areas in northwestern Iran.

## 6. Limitations

The present study had several limitations. First, only three seropositive cases were identified, limiting statistical power and preventing reliable analysis of associations with demographic variables such as age and sex. Second, serological findings could not be confirmed using additional parasitological, molecular, or imaging methods. Therefore, ELISA positivity should be interpreted cautiously, as it may indicate either previous or current exposure to *Fasciola* spp. Finally, the study population may not fully represent all humans at risk, potentially resulting in underestimation of seroprevalence.

## Author Contributions

F.R., Z.G.A., D.O.A.A., Y.A., M.Z.G., and S.Z. collected the sera and demographic data, and performed tha laboratory work. F.R. and Z.G.A. prepared the original draft of the manuscript. R.J. and K.D. supervised the research, edited the manuscript, and analyzed the data.

## Funding

This study was supported by Urmia University of Medical Sciences (10.13039/501100016286; 3038).

## Ethics Statement

All methods in the present study were conducted in accordance with the relevant guidelines and regulations of the Iran National Committee for Ethics in Biomedical Research. The study received approval from the Ethics Committee of Urmia University of Medical Sciences under the Ethical Code IR.UMSU.REC.1401.156. Participants were informed about the study, and a signed informed consent form was obtained.

## Consent

The authors have nothing to report.

## Conflicts of Interest

The authors declare no conflicts of interest.

## Data Availability

All data are presented in the manuscript.
